# Using general practice clinical information system data for research: the case in Australia

**DOI:** 10.23889/ijpds.v5i1.1099

**Published:** 2020-01-27

**Authors:** D Youens, R Moorin, A Harrison, R Varhol, S Robinson, C Brooks, J Boyd

**Affiliations:** School of Public Health, Curtin University, Perth, Australia; School of Population and Global Health, University of Western Australia; Swansea University Medical School, Singleton Park, Swansea, UK; Health Systems & Economics, School of Public Health, Curtin University; Department of Public Health, School of Psychology and Public Health, College of Science, Health and Engineering, La Trobe University

**Keywords:** General practice, administrative data, big data, health information systems, medical records system

## Abstract

General practice is often a patient’s first point of contact with the health system and the gateway to specialist services. In Australia, different aspects of the health system are managed by the Commonwealth Government and individual state / territory governments. Although there is a long history of research using administrative data in Australia, this split in the management and funding of services has hindered whole-system research. Additionally, the administrative data typically available for research are often collected for reimbursement purposes and lack clinical information.

General practices collect a range of patient information including diagnoses, medications prescribed, results of pathology tests ordered and so on. Practices are increasingly using clinical information systems and data extraction tools to make use of this information. This paper describes approaches used on several research projects to access clinical, as opposed to administrative, general practice data which to date has seen little use as a resource for research.

This information was accessed in three ways. The first was by working directly with practices to access clinical and management data to support research. The second involved accessing general practice data through collaboration with Primary Health Networks, recently established in Australia to increase the efficiency and effectiveness of health services for patients. The third was via NPS MedicineWise’s MedicineInsight program, which collects data from consenting practices across Australia and makes these data available to researchers.

We describe each approach including data access requirements and the advantages and challenges of each method. All approaches provide the opportunity to better understand data previously unavailable for research in Australia. The challenge of linking general practice data to other sources, currently being explored for general practice data, is discussed.

Finally, we describe some general practice data collections used for research internationally and how these compare to collections available in Australia.

## Introduction

Health systems produce large volumes of data and these data are increasingly created and stored in digital formats [1]. Such data can be an invaluable tool both for patient care and research. In Australia the administrative data generated in hospitals has supported research for decades [2], however general practice data has to date seen limited use as a research resource [3].

This paper adds to a limited body of evidence regarding the utility and availability of clinical general practice data for research in Australia. This is based on our experience of working with different sources of general practice data, which we have accessed through different approaches. We describe these approaches in terms of access, usage, challenges and linkage to other collections. We describe the approaches our research group is familiar with in detail, and provide an overview of other Australian data collections and leading examples internationally.

## Background

### The Australian Health System

General practices are entrenched in communities and are usually the initial interaction people have with the health system. While general practice provides health promotion, prevention, treatment and support; it is also the gateway to specialist services that support a growing population with chronic and complex comorbidities [4].

In Australia primary care and specialist services are provided largely by private providers who are reimbursed by the Commonwealth (Federal) Government on a fee-for-service basis [5]. Australia’s universal public insurance system, Medicare, ensures that all citizens and permanent residents have free access to public hospitals and reimburses general practitioners, specialists and other providers for services rendered (though providers may charge additional out-of-pocket costs [6]). It also provides reduced cost access to pharmaceuticals via the Pharmaceutical Benefits Scheme (PBS) [6]. In contrast, public hospitals in Australia are managed and operated by state and territory governments and are funded jointly by these state / territory governments and the Commonwealth Government. A significant recent development is the establishment in 2015 of 31 Primary Health Networks (PHNs) covering the country. These are not-for-profit companies independent of government, established to organise health services in their region. The main roles of the PHNs are to commission services to address gaps and meet prioritised local needs; to work closely with general practitioners and other health professionals to build capacity and the delivery of high quality care; and to integrate services at the local level to improve the patient experience and eliminate duplication [7]. Their key objectives are to improve the efficiency and effectiveness of medical services for patients, particularly those at risk of poor health outcomes, and to improve the coordination of care. The PHNs work with public and private services (including general practice) in their region but do not directly provide services [8].

### Administrative data supporting research in Australia

Administrative data regarding ambulatory care services (including general practice) and medication dispensations are routinely collected in Australia. The Medicare Benefits Schedule (MBS) captures data on all services rendered attracting reimbursement through Medicare [9] and hence includes almost all general practitioner contacts, many diagnostic tests, therapeutic procedures and specialist visits. PBS data include records of all medicines dispensed under the scheme [9]. These data are collected for reimbursement meaning that while they have very good coverage and quality, they often lack detail important for research. For example the PBS records medications dispensed with no information on prescribing, meaning important information needs to be estimated (for example dosage prescribed or the reason for prescription) and there is no information on unfilled prescriptions [10] (relevant if trying to understand, for example, primary non-compliance). The MBS records item numbers indicating reimbursable activities such as general practice consultations, procedures, the completion of pathology tests and so on [9, 11] without any information on context. For example, a record indicating a general practice consultation will not include information on the reason for the visit, the advice offered, or any condition(s) diagnosed. Similarly, a record may indicate the completion of a pathology test but will not hold any information on the results of that test, and in many cases may indicate the completion of any one of a range of tests, which attract the same reimbursement but are otherwise unrelated. Furthermore, where multiple pathology tests are ordered, only the three attracting the highest reimbursements are captured meaning a test which is relevant to a research question may not appear in MBS data. While these administrative data have certain strengths in their suitability for research (including completeness, whole population coverage, oversight by a single custodian) the limitations described, in particular the lack of clinical and patient information, make them unsuitable for many research questions.

### General practice data

Beyond this administrative data there is a substantial gap in the general practice data available for research in Australia [12]. Previously survey data has provided insight into general practice activity [13] although these surveys were not intended to facilitate whole-sector research linking with data from other sources or longitudinal follow-up of patients.

More suitable clinical data for many research questions is collected through the clinical information system (CIS) software increasingly used by general practitioners since their introduction in the 1990’s [14] to manage their patients [3]. This software initially focussed on collecting administrative and business related information, such as billing and scheduling, to assist with daily practice processes. These systems have expanded considerably to also collect clinical information including referrals, prescribed medications, development of patient management plans, pathology tests and other data. As the use of clinical information systems have increased over the past decade, tools to extract the data captured with these systems have been developed. These aid providers through clinical audits of patient cohorts and can highlight opportunities for improvement in business processes and patient care. These tools can extract de-identified patient information from clinical software and aggregate these data into measures related to patient visits, demographics, diagnoses, immunisations, pathology and so on. Many of these provide useful dashboard approaches and have been shown to enable quality improvement within general practice [15]. The development and use of these tools facilitates the extraction of clinical general practice data for research purposes, the focus of this paper.

### Development of data linkage in Australia

Data linkage capabilities within Australia are shaped by the division of health services [16]. Individual states have developed their own linkage centres over several decades and many of these have supported large numbers of research projects [17, 18]. These state-based systems link hospitalisation data with emergency records, disease registries, large surveys, community services and so on. These state-level data can be linked to MBS and PBS (Commonwealth) data on a project by project basis, though as such linkages require the release of linkable fields by one jurisdiction to another the approvals process becomes more complex. The Commonwealth Government established the Population Health Research Network (PHRN) in 2008 to provide Australian researchers with access to linkable de-identified data from a diverse and rich range of health datasets, across jurisdictions and sectors. Meanwhile the Australian Institute of Health and Welfare has been designated an Integrating Authority, meaning that it has the authority to undertake linkage including Australian (Commonwealth) Government data [19]. Through the PHRN Australia now has a dedicated capability for linkage of administrative and research data across all states and territories [20], though the approvals process for such linkages remains very challenging for researchers [21].

In summary, despite a strong tradition of linked data research in Australia using administrative (predominantly billing) data there are currently major limitations including the lack of information on the clinical management of patients in general practice, and obstacles to research using data from multiple sectors, making research capturing the patients journey through the health system challenging.

## Current approaches to accessing general practice data

Our research group currently accesses general practice data for research through three methods. The first involves working directly with individual practices. The second is through working with general practices in partnership with one of our researcher teams working with the Primary Health Networks (PHNs) in Western Australia. The third is via a centralised collection of general practice clinical information system data collected, maintained by and accessed via the MedicineInsight program run by NPS MedicineWise [22], which is an authority on the quality use of medicines in Australia. We describe the practicalities, benefits and challenges of each of these approaches.

### Working with general practices, directly and via Primary Health Networks

Access to general practice data for research relies in part on the CIS and associated data extraction systems, so a brief overview of these is warranted. As the clinical data collected in general practice has increased in scope, software vendors have developed their clinical information systems largely independently, without standardisation, resulting in a disparate array of methods to store and report information. For example, Best Practice and Medical Director, the two most common general practice management tools in Australia, use the incompatible medical terminology and health coding systems Pyefinch [23] / DOCLE (Doctor Command Language) [24] and the Medical Director termset, a derivative of SNOMED CT (Systematized Nomenclature of Medicine – Clinical Terms) [25]. Other applications such as PractiX, ZedMed and Genie use ICPC-2+ (International Classification of Primary Care) [23].

There is also diversity in the data extraction tools in use in Australia. The most popular is CAT4 (Clinical Audit Tool 4) developed by PenCS [26], which is provided to general practice through subsidised means by most (28 of 31) PHNs. Some PHNs provide POLAR GP (Population Level Analysis & Reporting) [27] as an alternative clinical auditing tool. Individual practices can select alternatives, with some choosing to use the Canning Tool [28] or to participate in the MedicineInsight program, which uses the GRHANITE (GeneRic HeAlth Network Information Technology for the Enterprise) extraction tool [29, 30] and a customised version of cdmNet.

#### Access to general practice data

Firstly, we describe experiences in accessing general practice data through working directly with practices.

There are some general steps that researchers can follow if wishing to access data from general practices. Ideally, the project should already have ethical approval from the researcher’s institution. Practices should be approached individually and invited to participate in the project. A brief information sheet is important, and practices may be invited to a workshop or information session where the researcher explains their project. For specific low-risk projects that have received ethics approval which require the participation of a cohort of patients (i.e. extraction of de-identified data), the Practitioner and the Practice Principal may consent on behalf of the patient depending on the practice’s registration form for new patients. Building trust with general practitioners is crucial for long-term data sharing. Regular sharing of data can be fostered where organisations or researchers are able to provide value to practices, through providing data summaries to practices either as in-person data summary reviews or via data dashboards or other visualisations. These approaches can help practices to increase data literacy and identify areas to target quality improvement activities.

#### Involvement of Software Vendors

Some recent developments in patient privacy [31] have prompted clinical software vendors to require oversight of how data captured via their software is used. A data application including the project description and evidence of ethical approval will usually be reviewed by the vendor’s committee, and the data request will usually incur a cost. Data extraction can occur in multiple ways. The simplest form of extraction is through predefined templates specific to each clinical software platform, though a unique extraction protocol is required for each platform as there is no agreed standard for extracting data. These templates are limited to what the vendor provides with requests for specific reports often associated with a financial outlay. Where these predefined templates are unsuitable, a customised extraction from the CIS database may be possible, though this requires SQL (Structured Query Language) expertise and governance approval from both the General Practice Principal (owner) and the software vendor. Software vendors are increasingly involved with data extraction requests by restricting data access to only general practice staff. It is becoming commonplace for researchers to work with CIS vendors to obtain data, as vendors want to ensure that data are being used in accordance with ethical approvals and to minimise risks of data breaches [31]. Accessing data this way therefore requires the research team to have sufficient technical skills to extract and store comparable data from across practices, substantial relationship and trust building with practices and administrative work to navigate approvals. These challenges will of course increase the more practices the research involves.

#### Involvement of Primary Health Networks

Where researchers need general practice data for their project the PHNs could (though do not necessarily need to) be approached. By involving the PHNs the researchers may get additional support if the project aligns with the PHNs health initiatives in the region. With the establishment of the PHNs there has been a renewed awareness of the importance and capability of data collected in general practice to improve both patient outcomes and operational efficiencies (i.e. workflows and practices). Prior to this, data extraction across the general practice continuum was sporadic and uncoordinated, making consistent quality improvement a challenge. Routine data extraction is now becoming commonplace in most general practices, with data extracts scheduled at a frequency and time identified by the practice and coordinated by the PHN and the extraction software vendor.

To ensure only approved individuals have access to the data, comprehensive terms of agreement and governance frameworks are defined and applied at both the practice and PHN. These facilitate the reciprocal exchange of data from general practice to PHNs in return for practice specific reports detailing comprehensive insights into practice performance that often include regional comparators. As secondary data custodians, the PHNs may, depending on the agreed governance, further use the aggregated information to help inform population health service planning and policy. Decisions relating to data access for research will depend on the nature of the research project. For example, an ecological analysis of a chronic disease, utilising data aggregated across patients before release from the practice, may be viewed differently from a project requiring longitudinal follow-up of patients and hence individual patient data. For data that are de-identified at the general practice, aggregated and shared with PHNs for continuous quality improvement, the Practice Principal will decide whether information is shared with the PHN.

#### Strengths of general practice data

Despite the immediate shortcomings of data collected in general practice, these can provide a diverse range of information to inform policy and practice which are not available elsewhere. The information collected in general practice, ranging from diagnostic, therapeutic, prescription, disease control data and so on, has the potential to complement the data generated in hospitals and elsewhere (e.g. allied health, aged care etc.) to provide a comprehensive view of the patient journey. When combined, these sources offer tremendous opportunities in improving, for example, prevalence and incidence estimates, health services evaluations, outcomes research and economic analyses.

Working with general practices via the means described above involves substantial work in building relationships to facilitate trust and the sharing of data (whether by researchers themselves or by the PHNs). Though this can involve a significant time investment, the building of these relationships can have the added advantage of facilitating the dissemination of research findings back into practices and hence informing the clinical care of patients.

#### Challenges in accessing data via general practices

The lack of standardisation in patient information management tools and data extraction tools used across general practice results in substantial variability and inconsistency in the data captured. The variability in the information captured by general practitioners, and between patient information systems / extraction tools in the type and coding of information, may not be an issue for individual practices using data for business improvement purposes but can be problematic for researchers aiming to use information aggregated across practices or make comparison between practices.

In addition to the technical aspects mentioned above, there is apprehension from health providers regarding the sharing of data with third parties, including de-identified data. This includes concerns about maintaining patient and provider privacy; a lack of confidence relating to information accuracy and completeness in the data collected; and, in our experience, an uneasiness in providing regulatory bodies with such data. This uneasiness follows examples such as the controversial Quality and Outcomes Framework (QOF) implemented in the United Kingdom (UK) [32] and Care.data which was abandoned over privacy concerns [33].

In the Australian context, the ability of the PHNs to provide data extraction software to practices and to provide snapshots of practice data in comparison to local or regional averages may encourage practices to share their data for quality improvement purposes. However, for individual researchers or research groups working in, for example, the university sector, challenges around data sharing may be more difficult to overcome. One of the advantages of partnering with the PHNs, where this is possible, is that many of the challenges outlined above may have been fully or partially resolved through the prior work of the PHNs. For example, most PHNs that have existing relationships with the practices in their region are providing snapshot reports back to practices, which may encourage data sharing and may have existing governance processes in place.

#### External linkages

With the exception of a few exploratory endeavours [34, 35] patient data are not typically linked between practices [35] or to other areas of the health system (e.g. tertiary care) [34] and, as such, the data collected by general practices in Australia cannot currently be used to fully understand patients’ interactions with the health system. International examples have demonstrated the power of using data from across the continuum of care in understanding, for example, associations between body-mass index and cancer [36], and risks of myocardial infarction and stroke following acute infection and vaccinations [37].

Although data linkage is not new, it is new to the field of general practice in Australia. The challenges described above in relation to data sharing also apply to data linkage, though given the reliance of data linkage on patient identifiers these issues can be more challenging to overcome. For example, a general practitioner or practice principal who is willing to provide access to de-identified data to support a research project may become much more hesitant where patient names and addresses are required to allow for linkage. Linkage may be facilitated through privacy preserving record linkage, whereby patient identifiers are irreversibly encoded prior to extraction from the practice, and linkage then performed on these encoded data [38]. Even with a technological solution such as this the steps of gaining the confidence and trust of practices remains vital, along with the necessity for a research team to have the technical skills to apply such methods. Of course, if the data are to be linked to hospitalisation or other data sources, separate applications will also need to be made to the custodians of those data collections which adds further complexity.

Obstructions to data sharing can be the result of legal or legislative barriers but are more often related to understanding the options around data sharing by data custodians. These barriers can often be dismantled by mitigating risks associated with the data sharing process through careful planning, secure protocols and legal agreements. Given the sensitivity of the information involved, and the growing desire to link a broader number of datasets, any risk mitigation models that facilitate broader sharing of data are valued by researchers, data owners and the public.

In cases where linkage to other data sources is desired, the patient is generally required to provide consent for linkage; although the National Health and Medical Research Council provide criteria under which an ethics committee may approve the use of patient data under a waiver of consent, considering the risk to patients and benefits of the research, practicality of obtaining consent for the given project, privacy protections and more [39]. Data linkage can be performed in Australia by linkage facilities under state [40] or Commonwealth Governments [19] or at university-based centres [41]; for a given project the party to perform the linkage will depend on the datasets being linked and the jurisdictions responsible for these data collections.

### NPS MedicineWise MedicineInsight Data

NPS MedicineWise was established twenty years ago in 1998 with the aim of promoting the quality use of medicines. The MedicineInsight program was established in 2011 as a quality improvement program to allow consenting general practitioners to assess their patterns of prescribing and patient care and to allow benchmarking at multiple levels [42]. This program involves practices signing up to share clinical data from their clinical information systems to MedicineInsight monthly, which allows MedicineInsight to provide insights into aggregated clinical data and provides practices the means to review their own activities. The extracted data includes information on patient demographics, reasons for encounters, conditions, prescriptions, observations, immunisation history and pathology tests, including results where available. Progress notes are not available. NPS MedicineWise has made MedicineInsight data available to external researchers to support primary health care research following ethical approval. As of July 2017 the program had recruited over 650 practices, which included information from over 3,300 general practitioners and 3.6 million regular patients [43].

#### Access

Data are accessed by application to NPS MedicineWise. Liaison staff including biostatisticians and epidemiologists work with researchers to discuss data requests. The release of data requires approval from MedicineInsight’s external independent Data Governance Committee. This committee includes general practitioners, consumer advocates, privacy experts and researchers. Practices enrol in the MedicineInsight program without the express consent of patients, though participating practices are provided with information to keep in waiting areas and patients may opt out of having their data reported from the practice to MedicineInsight.

#### Strengths

Practices are only enrolled if they use the patient management systems Medical Director or Best Practice. MedicineInsight staff perform data cleaning and coding of important information. This includes work to de-duplicate patients with multiple records at a single site, separate ‘clinical encounters’ from records that are administrative only and identify ‘active’ and ‘regular’ patients. Similarly, MedicineInsight apply geographical information such as remoteness and socioeconomic status using the Australian Statistical Geography Standard Remoteness Areas [44] and the Socio-Economic Indices for Areas [45], respectively. Researchers still need to do additional cleaning of their own as apparent errors remain in the data (for example, impossible values in various free-text fields including clinical observations in some cases). Clinical codes are used for some important fields including Anatomic Therapeutic Classification codes [46] being used for the medicines prescribed and the Logical Observation Identifiers Names and Codes (LOINC) system [47] which is used for pathology data.

A data book and a data dictionary are provided to aid interpretation of data [43, 48]. These provide background information on the collections, data elements (with explanatory notes), governance and ethics, data quality and some worked examples of how the data can be used. Although these are extensive, they lack some detail in comparison to data dictionaries that might be provided by data linkage branches with more substantial experience working with academic researchers. In some cases, the data book and data dictionary lack explanations on data that are automatically coded by the patient information management systems of the practices, such as some clinical observations and pathology requests and results. However, enough information and support is available to help researchers decide if this is a suitable source of data for their project.

Data include a scrambled patient identifier [22], allowing service, pathology, prescription and other data belonging to a single patient to be linked and importantly, allowing for patients to be followed longitudinally. The program has been running since 2011 and, once a practice joins, the full clinical history of patients is available. Information on representativeness of the patient population in the MedicineInsight data in comparison to the general Australian population is provided for some important fields [48].

MedicineInsight provides some derived variables on request, notably including patient diagnostic flags for a number of important chronic conditions. These are likely to be useful to many researchers whether aiming to understand prevalence within a population of interest, prescribing within specific cohorts or countless other research questions. While the generated diagnostic flags may not always reflect the cohort a researcher is interested in or the level of detail needed (e.g. a researcher may need the date a diagnosis was made rather than a flag of its presence), detail of the data available gives researchers a great deal of flexibility in constructing their own cohorts and indicators.

#### Challenges

As NPS MedicineWise seeks to recover the cost of data provision, researchers are required to pay data access fees, which may present a barrier for some researchers, although this is typical for any researcher wishing to access an existing dataset.

Most of the limitations of MedicineInsight data reflect the fact that it is a collection of the data generated across millions of encounters at hundreds of practices. Inconsistencies in the data captured and / or coded by general practitioners, or at any other step of the data generating process prior to MedicineInsight receiving the data, will ultimately be reflected in the data provided to researchers.

Some important data are provided as free-text fields, for example patient diagnoses and reason for encounter. This can result in a substantial time investment for researchers to identify clinical cohorts of interest, particularly when compared to collections such as hospitalisation data where this information is provided as International Classification of Disease (ICD) codes. The possibility of spelling errors and incorrect use of fields by general practitioners, for example the diagnosis field being used to record symptoms or family histories, can add to the time required to clean the data. This does, however, provide researchers with a high level of control over how they use the data. MedicineInsight do also offer a service of providing cohorts of interest which may save researchers significant time, albeit with added cost.

There are issues in some cases with missing data, for example, pathology test results with no unit of measurement recorded (approximately 8% of tests in the data checked by the authors) and prescription records with no information on medicine strength (approximately 1% of prescriptions) and no Anatomic Therapeutic Class code (4% of records), issues which ultimately reflect the recording of information by general practitioners.

As the MedicineInsight program staff continue to work with both general practices and researchers the data available and supporting documentation will continue to improve.

#### Linkage to other data sources

Linkage of MedicineInsight data is currently in its infancy, both in terms of linking general practice data between practices and linking to other data collections. Identifying information such as name, date of birth and address is not collected by MedicineInsight and data linkage can only be implemented using encoded versions of person-identifying information. These cryptographic hashes of the person-identifying information are performed within the practice so no identifying information leaves the practice [22]. Linkage is being explored using the GRHANITE key and Statistical Linkage Key (SLK) matching algorithms. Research is occurring in Victoria to link MedicineInsight data with cancer registry data. The issue of patient identifiers that do not link between practices, meaning that patients who visit multiple providers will appear multiple times in the data, has been explored using algorithms by the University of Melbourne. Longitudinal research is possible for patients who receive their care through a single practice. MedicineInsight are planning work to identify records across practices belonging to the same patient, which will facilitate improved longitudinal research.

## Comparisons to other systems

We present here brief comparisons to other relevant general practice data collections. We describe other Australian collections that we are aware of; and provide comparisons to the UK’s Clinical Practice Research Datalink (CPRD) and Wales’ Secure Anonymised Information Linkage (SAIL) databank, which represent ‘gold standards’ in general practice data collections. We also describe systems in Canada at a similar level of development to the approaches described above. Canadian examples provide useful points of comparison on account of the two countries similar federations, and similarities in access to both public hospitals and medical services [49, 50].

### Australian examples

The Melbourne East Monash General Practice Database (MAGNET) presents another collection of general practice data to support research in Australia [51]. This database includes practices within a single region in Melbourne. Practices which agree to participate have their data encrypted and extracted to a data warehouse (without identifying information) where it can then be used for research. Data are fed back to practices for quality improvement, which is the primary purpose of the underlying infrastructure. The data include patient demographics, episodes of care, diagnoses, medications prescribed, observations, investigations ordered and received, immunisations and more, while some practice data is also included. This collection is much smaller than MedicineInsight in terms of practice and patient numbers included. The MAGNET database includes a SLK which allows the same patient to be identified at different practices and hence duplication avoided and may also support linkage to external data collections. MAGNET has been used as the basis for research investigating service use [52], prescribing [53], cohort characterisation [54] and more.

More recently, the Data for Decisions project has been established through the University of Melbourne [55]. This program has been recruiting practices since only 2017 and is described as being in its start-up phase. De-identified data are transferred to a data repository using GRHANITE, and the program may facilitate linkage to different datasets. The data extracted appears comparable to other data collections; with initial projects assessing antimicrobial prescribing, chronic disease programs and disease detection [56]. Data is accessed following approval by an independent Data Governance Committee, and a practice opt-in, patient opt-out model has been adopted.

Australia’s earliest collection of CIS data is the General Practice Research Network, administered by the Health Communication Network, publishers of Medical Director. This system extracts information on medications, conditions, history, imaging and pathology tests ordered, observations taken, basic patient demographics and risk factors [57]. This repository is restricted to practices which use a single CIS (Medical Director) and operates on a GP opt-in, patient opt-out model. The dataset includes information for approximately 3 million unique patients of 1,100 GPs [58]. Since 1999 this network has supported publications examining prescribing and vaccination [59], though publications using this resource appear to be rare, particularly in the last decade.

### International examples

Internationally, one of the most comprehensive sources of primary care data used for research is the Clinical Practice Research Datalink (CPRD) in the UK [60]. The CPRD has provided data and services to support research investigating pharmacovigilance and the use of medicines, informing of health policy and healthcare delivery and exploring disease risk factors for more than thirty years. The CPRD is a primary care database of de-identified medical records from general practices in the UK including over 11 million registered patients split across two datasets [61]. The CPRD has been designed to provide a representative population dataset and is linked to a number of other sources to provide a rich data resource. The CPRD includes information on encounters, immunisations, tests, therapies and patient socio-demographics. Data including diagnoses are recorded using version 2 Read codes. One of the major strengths of the CPRD is linkage to external data sources including hospitalisation, mortality and disease registries for a subset (over half) of practices [60]. Furthermore, the CPRD has a broad ethical approval for observational research using the primary care data and established linkages simplifying data access. Referrals to secondary care, and information fed back from secondary care are also included. Data quality is promoted through the Quality and Outcomes Framework in place in the UK which provides financial incentives for recording of important data items [62]. Similar financial incentives are currently being planned in Australia, which will reimburse practices for participating in quality improvement practices and sharing a minimum dataset demonstrating this with their local PHN [63], however the implementation of these incentives has been subject to delays and it is as yet unknown how data recording and quality may change as a result of the incentives [64].

The SAIL (Secure Anonymised Information Linkage) databank is based in Swansea University Medical School. SAIL works in partnership with researchers and health professionals, aiming to maximise the value of routinely collected individual level data through record linkage and to enable and support health related research [65]. Linked data from different sources is created by the NHS (National Health Service) Wales Informatics Service using the NHS number. The Welsh Demographic Service [66] provides personal information of all persons who have registered with a general practice or received care from health services in Wales [67, 68]. SAIL links a wide range of data including general practice, hospitalisation, national screening programmes, the national cancer registry and more [65]. The primary care dataset contains information on patient encounters with primary care; capturing the signs, symptoms, test results, diagnoses, prescribed treatment, specialist referrals and social aspects relating to the patient’s home environment [69]. Similar to NPS MedicineInsight, SAIL recruits general practices to voluntarily share data; practices sign up to share data without the express consent of patients, though practices are provided with information to keep in waiting areas and patients may opt out of having their data reported to SAIL. As of November 2018, SAIL had recruited 334 general practices (76% of all Welsh practices) which relates to information for over 2.5 million patients. SAIL has now been used to support a range of research including follow-up of clinical trials [70], evaluation of medication use [71], epidemiological studies [72] and policy evaluations [73].

Currently the general practice data available for research in Australia is comparable to the CPRD and SAIL systems in some ways, including the breadth of data captured, the centralised data application and practice opt-in / patient opt-out consent model. The key point on which these UK systems are further developed than any system in Australia is the availability of linkage between general practice and other data sources, though this capability is developing with regards to the MedicineInsight data collection and other collections within Australia.

There are also examples of developing primary care linkages in Canada. These include the Electronic Medical Record Administrative Data Linked Database (EMRALD) in Ontario, and the Canadian Primary Care Sentinel Surveillance Network (CPCSSN), which includes practices across most provinces. Like MedicineInsight, these programs recruit general practices to voluntarily report data, based on their use of CISs. Each of these captures some information on patient encounters with physicians, medications prescribed, laboratory investigations and so on. The EMRALD database includes linkage to other health related administrative, survey, registry, demographic data and more through the comprehensive data holdings of the Institute for Clinical and Evaluative Sciences [74]. This provides a more complete picture of patient’s care than is possible through the primary care data collections in Australia, although the system is confined to a single Canadian province [75]. The CPCSSN is larger than EMRALD in the number of practices and patients captured, with over 200 practice sites and 1.5 million patients as at May 2016 [76] though is smaller than the MedicineInsight collection on these measures. Furthermore, the CPCSSN has recently begun to be used for research projects linking primary care data to other sources such as hospitalisation and census data [76, 77]. Case definitions are applied for a number of chronic conditions, similar to the MedicineInsight collection, though in the case of the CPCSSN these are supported by the use of ICD-9 codes in Canadian CIS data. The scope of the data collected is otherwise similar. One problem common to both the CPCSSN and general practice data in Australia, is that when patients visit multiple providers they will exist in the database more than once and duplicated patients cannot be differentiated.

## The road ahead

Despite the increased use of CISs in general practice, and extensive collections of administrative data, research covering the whole health system has been hampered by jurisdictional issues obstructing linkages [3, 21], and until relatively recently, the lack of any centralised collections of general practice data. Such collections are now becoming available, and technological advances are developing to help resolve challenges around sharing of patient data for linkage to other sources. Even with technological solutions available, it remains important to build trust among general practitioners that any patient data shared remain confidential and that evidence generated is fed back to practices to support patient care and practice processes. Though evidence on the Australian public’s views on data use is scarce, opinion polls suggest that a vast majority are supportive of health records being used for research [78]. Meanwhile most GPs are supportive of general practice research though there are barriers to involvement [79]. Data sharing models that maintain patient and provider privacy are therefore likely to be valued by all. These data sharing challenges were the focus of an Australian Productivity Commission inquiry (under the Productivity Commission Act 1998) into the benefits and costs of options for increasing the availability and use of public and private sector data by individuals and organisations. The resulting report prepared by the Productivity Commission [80] proposes a new legal and policy framework to allow public and private sector data to flow. The recommendations in the report provide good foundations to build future data sharing and data linkage models.

## Conclusion

Within Australia there is extensive population-level administrative data captured on the use of health and other services. However, the delivery of services by different levels of government, and hence the holding of data by different levels of government, presents challenges to researchers aiming to perform whole of system research. Furthermore, administrative data covering primary care services are generally limited to basic information gathered for reimbursement purposes. Where research questions require the use of more detailed primary care data including patient diagnoses, the ordering of and results of pathology testing and measurement of observations, the prescribing of medications and so on, there are limited options available to researchers. We have described three avenues to access this data from general practices, though there are limitations to each of these methods. One limitation of the approaches described here is the limited ability to link the detailed clinical data from general practices to other sources of information such as hospital admissions and even other general practices although this capacity is emerging.

International examples demonstrate that comprehensive individual-level data capturing primary, secondary and tertiary care can be linked and made available to researchers, though these countries have universal unique identifiers for patients. We hope that the data available to Australian researchers can continue to improve, following these examples.

## Ethics statement

As this is a commentary paper, ethical approval is not required.

## Abbreviations

AIHW	Australian Institute of Health and Welfare
BEACH	Bettering the Evaluation and Care of Health
CAT4	Clinical Audit Tool 4
cdmNet	Chronic Disease Management-Net
CHeReL	Centre for Health Record Linkage
CIS	Clinical Information System
CPCSSN	Canadian Primary Care Sentinel Surveillance Network
CPRD	Clinical Practice Research Datalink
DOCLE	Doctor Command Language
EMRALD	Electronic Medical Record Administrative Data Linked Database
GP	General Practitioner
GRHANITE	GeneRic HeAlth Network Information Technology for the Enterprise
ICD	International Classification of Diseases
ICPC	International Classification of Primary Care
LOINC	Logical Observation Identifiers Names and Codes
MAGNET	Melbourne East Monash General Practice Database
MBS	Medicare Benefits Schedule
NWIS	NHS Wales Informatics Service
NHS	National Health Services
NPS	National Prescribing Service
PBS	Pharmaceutical Benefits Scheme
POLAR	Population Level Analysis & Reporting
PHN	Primary Health Network
PHRN	Population Health Research Network
QOF	Quality and Outcomes Framework
SAIL	Secure Anonymised Information Linkage
SLK	Statistical Linkage Key
SNOMED	Systematized Nomenclature of Medicine
SQL	Structured Query Language
